# Clinical and multimodal biomarker correlates of ADNI neuropathological findings

**DOI:** 10.1186/2051-5960-1-65

**Published:** 2013-10-09

**Authors:** Jon B Toledo, Nigel J Cairns, Xiao Da, Kewei Chen, Deborah Carter, Adam Fleisher, Erin Householder, Napatkamon Ayutyanont, Auttawut Roontiva, Robert J Bauer, Paul Eisen, Leslie M Shaw, Christos Davatzikos, Michael W Weiner, Eric M Reiman, John C Morris, John Q Trojanowski

**Affiliations:** 1 Department of Pathology & Laboratory Medicine, Institute on Aging, Center for Neurodegenerative Disease Research, University of Pennsylvania Perelman School of Medicine, Philadelphia, PA, USA; 2 Departments of Neurology and Pathology & Immunology, Washington University School of Medicine, St. Louis, MO, USA; 3 Section of Biomedical Image Analysis, Department of Radiology, and Center for Biomedical Image Computing and Analytics, University of Pennsylvania, Philadelphia, PA, USA; 4 Banner Alzheimer's Institute, 901 East Willetta Street, Phoenix, AZ, USA; 5 University of California at San Diego, San Diego, CA, USA; 6 Center for Imaging of Neurodegenerative Diseases, Department of Radiology, San Francisco VA Medical Center/University of California San Francisco, San Francisco, CA, USA

**Keywords:** Alzheimer’s disease, Mild cognitive impairment, CSF, MRI, Autopsy, Neuropathology, Dementia, Biomarkers, Amyloid, Tau

## Abstract

**Background:**

Autopsy series commonly report a high percentage of coincident pathologies in demented patients, including patients with a clinical diagnosis of dementia of the Alzheimer type (DAT). However many clinical and biomarker studies report cases with a single neurodegenerative disease. We examined multimodal biomarker correlates of the consecutive series of the first 22 Alzheimer’s Disease Neuroimaging Initiative autopsies. Clinical data, neuropsychological measures, cerebrospinal fluid Aβ, total and phosphorylated tau and α-synuclein and MRI and FDG-PET scans.

**Results:**

Clinical diagnosis was either probable DAT or Alzheimer’s disease (AD)-type mild cognitive impairment (MCI) at last evaluation prior to death. All patients had a pathological diagnosis of AD, but only four had pure AD. A coincident pathological diagnosis of dementia with Lewy bodies (DLB), medial temporal lobe pathology (TDP-43 proteinopathy, argyrophilic grain disease and hippocampal sclerosis), referred to collectively here as MTL, and vascular pathology were present in 45.5%, 40.0% and 22.7% of these patients, respectively. Hallucinations were a strong predictor of coincident DLB (100% specificity) and a more severe dysexecutive profile was also a useful predictor of coincident DLB (80.0% sensitivity and 83.3% specificity). Occipital FDG-PET hypometabolism accurately classified coincident DLB (80% sensitivity and 100% specificity). Subjects with coincident MTL showed lower hippocampal volume.

**Conclusions:**

Biomarkers can be used to independently predict coincident AD and DLB pathology, a common finding in amnestic MCI and DAT patients. Cohorts with comprehensive neuropathological assessments and multimodal biomarkers are needed to characterize independent predictors for the different neuropathological substrates of cognitive impairment.

## Background

Studies based on clinical and neuropathological diagnoses have shown that Alzheimer’s disease (AD) is the most common cause of dementia [1-4]. However, there are several neurodegenerative [5-8] and non-neurodegenerative pathologies [1,9-12] that are known to contribute to cognitive impairment and a dementia diagnosis. Different clinical dementia syndromes have degrees of clinico-pathological correlation, therefore if clinical diagnosis is used to estimate the accuracy of biomarkers and their cutoffs, inaccurate results may occur [13-15]. In addition, coincident neurodegenerative diseases (NDDs) and vascular pathology are common findings in subjects with AD in autopsy series [1,9,14,16-18]. However, clinical studies of dementia of the Alzheimer type (DAT) and other NDDs assign a single primary clinical diagnosis to patients. Accordingly, most biomarker studies are based on clinical diagnoses and report results on subjects using a single NDD diagnosis as the outcome. While the use of clinical diagnoses is helpful for the screening and the evaluation of new biomarkers, substantial follow up studies are needed to establish the performance of these biomarkers. Such studies should include in the analysis consecutive series of patients with NDDs and non-NDDs together with multimodal biomarkers assessing their performance in complex settings with several coincident diseases. Previously, retrospective studies have analyzed the correlation between neuropathological findings and CSF [14,19,20], magnetic resonance imaging (MRI) [21-24] and positron emission tomography (PET) [21,25-27]. Most of these studies tried to categorize patients into a single diagnostic category. Here, we instead tried to assess how different combinations of biomarkers can detect different coincident pathologies and therefore predict the different combinations of neuropathological substrates of the cognitive impairment in the studied subjects to help identify homogeneous cohorts of patients for clinical studies and clinical trials. This is especially true of clinical trials for DAT in which one pathology is targeted for study such as therapies that target Aβ or tau mediated mechanisms of neurodegeneration. Thus, here we specifically tested cerebrospinal fluid (CSF) biomarkers for the diagnosis of AD (Aβ_1-42_, total tau (t-tau) and phosphorylated tau (p-tau_181_) and dementia with Lewy bodies (DLB) (α-synuclein), MRI hippocampal and occipital pathology for the diagnosis of coincident DLB and medial temporal lobe (MTL) pathologies and occipital hypometabolism for the diagnosis of DLB. In addition, we tested the neuropathological association of hallucinations, memory and executive dysfunction. For this study, we examined the first 22 patients in the Alzheimer’s Disease Neuroimaging Initiative (ADNI) who were longitudinally followed to death and underwent postmortem examination.

## Methods

### Participants and neuropsychological testing

Data used in the preparation of this article, was downloaded from the ADNI database August 19th 2013. The ADNI was launched in 2004 by the National Institute on Aging (NIA), the National Institute of Biomedical Imaging and Bioengineering (NIBIB), the Food and Drug Administration, private pharmaceutical companies and non-profit organizations as reviewed elsewhere [28] (see additional information in http://www.adni-info.org and Additional file 1: Table S1). Here, we focused on the first 22 ADNI subjects who came to autopsy, i.e. 21 from ADNI 1 and one subject from ADNI 2 (Table 1 and Additional file 1: Table S3). A diagnosis of MCI was established as previously described [29,30] and DAT was based on the National Institute of Neurological and Communicative Disorders and Stroke–Alzheimer’s Disease and Related Disorders Association criteria for probable AD [29,31]. Summary composite executive and memory measures developed by Gibbons et al. [32] and Crane et al. [33], respectively, were used to estimate the cognitive profile differences associated with the neuropathological diagnoses.

**Table 1:** Subject characteristics

	**AD**	**AD + MTL pathology**	**AD + DLB**	**AD + DLB + MTL pathology**	**p-value**
	**n = (7)**	**n = (5)**	**n = (6)**	**n = (4)**	
Longitudinal clinical diagnosis	1 CN to MCI	1 MCI stable	1 MCI to DAT	2 MCI to DAT	-
	2 MCI stable	3 MCI to DAT			
		1 DAT	5 DAT		
				2 DAT	
	2 MCI to DAT				
	2 DAT				
Neuropathological diagnosis	1 LNC AD	1HNC AD + MTL-TDP	1 HNC AD + DLB	1 DLB-LNC	-
	3 HNC AD			AD + AGD	
	3 HNC AD + SVD-I	2 LNC AD + AGD + MTL-TDP	3 HNC	1 DLB- LNC AD + MTL-TDP	
				2 HNC AD + DLB + MTL-TDP	
		1HNC AD + AGD + MTL-TDP + HS			
		1HNC AD + AGD + MTL-TDP + HS + SVD-I			

			AD- + DLB		
			1 HNC AD-DLB + SVD-I		
			1 DLB + LNC AD		
Age at death (years) ^2^	80 (77–83)	86 (82–88)	80.5 (72.25-83.75)	81 (77.75-84.75)	0.41
Gender (n male/total)	3/7	4/5	6/6	4/4	1.0
Education (years) ^1^	15.4 (2.4)	15.0 (2.4)	16.1 (2.6)	14.0 (2.3)	0.62
Baseline visit to death (weeks) ^2^	240.7 (98.7-256.8)	181.9 (108.4-289.0)	136.5 (80.9-278.3)	234.9 (189.1-257.1)	0.72
APOE ϵ4 (n positive/total)	4/7	1/5	5/6	1/3	0.18
ADAS-Cog (13 item) baseline ^2^	20.0 (10.3-28.0)	22.0 (17.7-29.0)	33.3 (18.0-54.7)	30.3 (25.3-35.0)	0.041
Aβ_1-42_ (pg/mL) ^2^	134.0 (86.0-261.0)	249.0 (123.0-261.0)	138.0 (82.0-152.0)	171.0 (134.0-201.9)	0.59
T-Tau (pg/mL) ^2^	141.5 (60.0-274.0)	65.0 (55.0-89.0)	88.0 (37.0-154.0)	73.0 (56.0-103.6)	0.61
P-Tau_181_ (pg/mL) ^2^	51.0 (17.0-70.0)	22.0 (12.0-33.0)	28.0 (11.0-45.0)	21.0 (19.0-24.0)	0.32

^1^Mean (Standard Deviation); ^2^Median (Minimum-Maximum).

AGD: Argyrophilic grain disease; SVD-I: Small vessel disease (arteriolosclerosis and/or cerebral amyloid angiopathy) and one or more infarcts); HNC AD: High neuropathologic change Alzheimer’s disease; HS: Hippocampal sclerosis; LNC AD: Low neuropathological change Alzheimer’s disease; MTL-TDP: TDP pathology circumscribed to the medial temporal lobe.

### CSF biomarker collection and analysis

Baseline CSF samples were obtained using polypropylene collection and transfer tubes in the morning after an overnight fast and processed as described (Additional file 1) [34,35]. Aβ_1-42_, t-tau, and p-tau_181_ were measured using the multiplex xMAP Luminex platform (Luminex Corp, Austin, TX) with Innogenetics (INNO-BIA AlzBio3; Ghent, Belgium; for research use–only reagents) immunoassay kit–based reagents. The capture monoclonal antibodies used were 4D7A3 for Aβ_1-42_, AT120 for t-tau and AT270 for p-tau_181_. The analyte-specific detector antibodies were HT7, for tau, and 3D6, for the N-terminus of Aβ (immunoassay performance details described in Shaw et al. [35] and Additional file 1). For the α-syn assay, Luminex MicroPlex Microspheres (Luminex Corp, Austin, TX) were chemically coated with rabbit anti-α-syn antibody ASY-1 and biotinylated goat anti-human α-syn antibody (R&D systems, Minneapolis, MN, USA) was used as the detection antibody [36,37].

### Magnetic resonance imaging acquisition and processing

Acquisition of 1.5-T MCI data at each performance site for the 21 ADNI 1 subjects followed a previously described standardized protocol that included a sagittal volumetric 3D MPRAGE with variable resolution around the target of 1.2 mm isotropically. The scans subjected to several correction methods including gradwarp, B1 calibration, N3 correction, and (in-house) skull-stripping (see http://adni.loni.usc.edu/ and [38] for further details). The images were processed with a freely-available pipeline [39] (for software, see http://www.rad.upenn.edu/sbia). Briefly, images were segmented into 3 tissue types: GM white matter (WM), and CSF. After a high-dimensional image warping to an atlas, regional volumetric maps for GM, WM and CSF were created, referred to herein as RAVENS maps. RAVENS maps are used for voxel-based analysis and group comparisons of regional tissue atrophy, as well as for constructing an index of AD brain morphology.

### FDG-PET acquisition

FDG-PET data was acquired and reconstructed with the use of measured-attenuation correction and the specified reconstruction algorithm for each scanner type according to a standardized protocol (http://adni.loni.usc.edu/data-samples/access-data/). All images were pre-processed by ADNI PET Coordinating Center investigators at the University of Michigan and uploaded to the LONI ADNI website. These images were downloaded by investigators at Banner Alzheimer’s Institute for additional pre-processing using SPM5 (http://www.fil.ion.ucl.ac.uk/spm) for computation of the HCI, for voxel-wise group comparison and by the investigators at U. Penn for the primary FDG-PET measure of left/right occipital regions.

HCI is a summary measure that reflects how the pattern and magnitude of cerebral hypometabolism in an individual’s FDG-PET image corresponds to that in probable AD patients. It was computed as previously described [40]. In addition to this global single index and as our primary FDG-PET measure, the relative cerebral metabolic rate for glucose (CMRgl) from anatomically predefined left and right occipital regions of interest (ROIs) was also extracted with global counts as reference region. Group comparisons were carried out also using a voxel-wise two-sample independent t-test implemented in SPM5 accounting for the whole brain PET counts variations using proportional scaling.

### Neuropathological procedures and diagnosis

The ADNI Neuropathology Core (ADNI-NPC) was funded and established at Washington University in St. Louis in September 2007; therefore no autopsies were performed on deceased ADNI subjects prior to this time. Established procedures for obtaining informed consent for autopsy and the autopsy procedures has been previously described [41]. Briefly, participating centers undertake their own brain assessment and provide standard sets of fixed tissue blocks/sections and frozen sections to the ADNI-NPC or send the brain to the ADNI-NPC if the center does not routinely perform neuropathological assessments. Formalin-fixed paraffin embedded tissue blocks are obtained from the left cerebrum for the following 16 areas: middle frontal gyrus, superior and middle temporal gyri, inferior parietal lobe (angular gyrus), occipital lobe to include the calcarine sulcus and peristriate cortex, anterior cingulate gyrus at the level of the genu of the corpus callosum, posterior cingulate gyrus and precuneus at the level of the splenium, amygdala and entorhinal cortex, hippocampus and parahippocampal gyrus at the level of the lateral geniculate nucleus, striatum (caudate nucleus and putamen) at the level of the anterior commissure, lentiform nucleus (globus pallidus and putamen), thalamus and subthalamic nucleus, midbrain, pons, medulla oblongata, cerebellum with dentate nucleus and spinal cord when available.

Sections from the different blocks were stained using hematoxylin and eosin and a modified Bielschowsky silver impregnation; immunohistochemistry (IHC) was performed using antibodies to ubiquitin (Dako, Carpinteria, CA), phosphorylated tau (PHF1, a gift from Dr. P. Davies, Albert Einstein College of Medicine, Yeshiva University, NY), Aβ (10D5, Eli Lilley, Indianapolis, IN), phosphorylated α-synuclein (Wako, Richmond, VA), and phosphorylated TDP-43 (Cosmo Bio, Carlsbad, CA) using methods previously described [41]. The neuropathological diagnosis for each case was determined in accordance with previously described criteria [7,42] for the pathological diagnosis of AD using the diagnostic nomenclature of the National Alzheimer’s Coordinating Center for diagnostic neuropathology [43] (DAT refers to the clinical diagnosis, whereas AD refers to the neuropathological diagnosis in this manuscript). Notably, this allows for a single primary neuropathological diagnosis and any other coincident secondary neuropathological diagnoses in addition to the primary neuropathological diagnosis [43]. In addition, Braak neurofibrillary tangle (NFT) staging [44], Consortium to Establish a Registry for AD (CERAD) scores [45], semiquantitative ratings for neuritic and diffuse plaques, Lewy body disease stage and the probability of DLB [46] were recorded. The Cairns et al. [47] and Mackenzie et al. [6] criteria and the National Institute on Aging-Alzheimer’s Association guideline [48] were applied for the diagnosis of frontotemporal lobar degeneration (FTLD) and AD, respectively. Due to the small number of cases and the heterogeneity of diagnoses, we grouped HS, AGD and MTL-TDP deposition under the category MTL pathologies. In addition, five cases had a pathological diagnosis of SVD-I.

### Statistical analysis

For the comparison of the four diagnostic groups listed in Table 1 Kruskall-Wallis test was applied to non-normally distributed variables and ANOVA was applied for those with a normal distribution. For further analyses, a Box-Cox transformation was applied to non-normally distributed variables and parametric tests were applied. To analyze association between two quantitative variables, Pearson correlation coefficient was used. Braak stages were dichotomized into a low (I/II) and high (V/VI) group. A logistic regression model was applied for multivariable analyses in case of a binary dependent variable (DLB positivity, e.g.) or a linear regression in case of quantitative dependent variables. Cutoffs for classification models were selected to maximized sensitivity and specificity. All statistical tests were two-sided. Most of our analyses were specified *a priori* based on previous findings (occipital hypometabolism in DLB and differences in hippocampal volume) and were not corrected for multiple comparisons. For multiple comparison adjustment of tests not based on an *a priori* hypothesis, the Holmes correction was used for non-imaging (i.e. for the cognitive tests) or non voxel-based imaging data. Statistical significance was set at the *p* < 0.05 level. For secondary voxel-wise analysis, uncorrected p = 0.005 was used.

## Results

### Brain donation status in ADNI and ADNI autopsy rates

Information about the participation in the brain donation program is available on a total of 1119 ADNI subjects (who were recruited in ADNI-1, ADNI-GO and ADNI-2): 653 subjects have made a decision to donate their brains at death, 5 are reviewing the information and 461 have not made a decision. Of the 653 who have made a decision, 139 (21.1%) are not participating in the autopsy program. The main reason not to take part in this brain donation program was the unwillingness of the subjects in 59 cases, followed by the lack of logistics in the study site in 31 cases, the participation of the patient in another autopsy program in 15 cases, the inconvenience and burden for the family in 9 cases and religious reasons in another 9 cases. Of the 514 cases who agreed to participate, 327 signed the provisional consent, 61 are reviewing the form and for 126 subjects the form will be completed at time of autopsy. Finally, 285 of the 461 subjects who have not made a decision have been given information about the program and 176 have not been approached. Up to the data download time on 17 August, 2013 there were 61 known deceased ADNI subjects, although deaths that occurred in subjects lost to follow-up is not always known. We compared the ADNI 1 subjects who died and came to autopsy to those who died with an autopsy and those that remain alive (or were lost to follow-up and no information about death is available). Dead patients who died without autopsy or remained alive were less cognitively impaired that those that came to autopsy (Additional file 1: Table S2).

### Neuropathological findings

**Figure 1:**
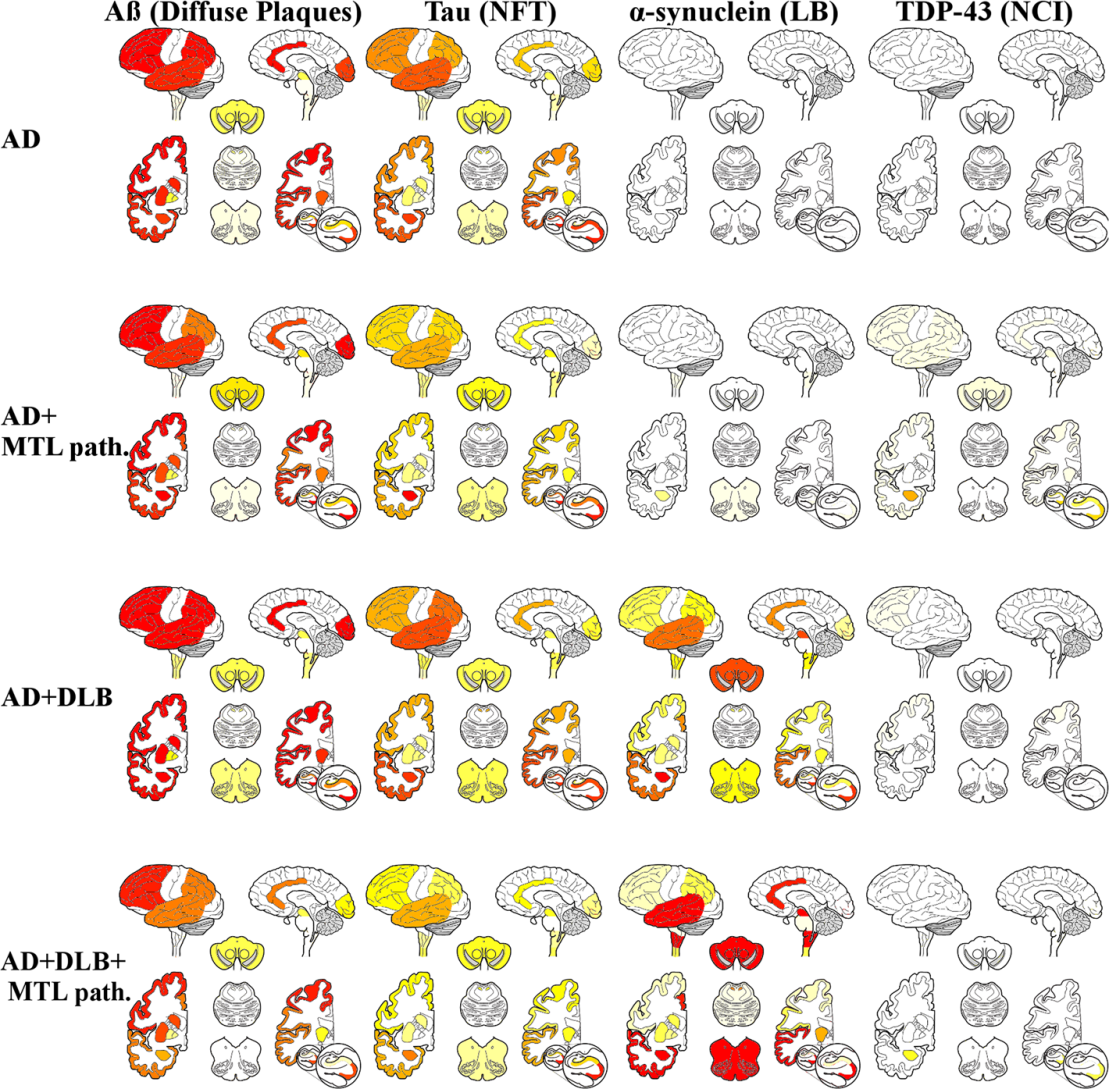
Heatmaps summarizing the semiquantitative neuropathological grading (from left to right: diffuse amyloid plaques, neurofibrillary tangles (NFT), Lewy bodies (LB) and neuronal citoplasmatic TDP-immunoreactive inclusions (NCI)) for the different neuropathologic diagnostic groups (from top to bottom: AD, AD + MTL pathology, AD + DLB, AD + DLB + MTL pathology and DLB + MTL pathology).

The primary neuropathological diagnosis was AD for 19 cases and DLB for 3; all 22 cases met diagnostic criteria for AD, including 12 who were clinically diagnosed with mild cognitive impairment (MCI) due to AD (Table 1). However, only 4 cases had a single AD neuropathological diagnosis, whereas all other cases had two or more neuropathological diagnoses, with AD and DLB being the most common comorbidities (10 cases). Other coincident diagnoses included: small vessel disease with infarcts (SVD-I), argyrophilic grain disease (AGD), MTL-TDP and hippocampal sclerosis (HS). In addition, 5 cases had coincident SVD-I. The TDP pathology was confined to amygdala, entorhinal cortex and dentate gyrus in all AD+DLB+MTL-TDP cases. In the AD + MTL pathology group TDP pathology was confined to amygdala, entorhinal cortex and dentate gyrus in two cases, one case had a low degree of TDP pathology in CA 1 region and the last case had a low degree of TDP pathology in CA1 and neocortical regions. For the purpose of this study subjects were grouped into AD and AD + DLB (irrespective of which one was the primary diagnosis). These groups were further stratified based on the absence or presence of additional comorbidities affecting the MTL as summarized in Table 1. All cases except one AD + DLB case (without any cerebral amyloid angiopathy) had mild or moderate cerebral amyloid angiopathy. Four cases in the AD group had severe atherosclerosis whereas other cases had none or mild atherosclerosis. A detailed neuropathological description of all 22 cases can be found in Additional file 1: Table S3 and ‘heatmaps’ that summarize the neuropathological findings in the different groups are displayed in Figure 1. The only clinical difference between groups was a greater cognitive impairment as measured by the ADAS-Cog in the AD + DLB and AD + DLB + MTL-TDP groups compared to the AD and AD + MTL-TDP groups (Table 1).

#### Clinical findings

At baseline, there were one cognitively normal subject (CN), eleven MCI and ten DAT patients. At the last visit prior to death, the CN subject had converted to MCI and eight MCI subjects had converted to DAT. All 18 subjects who died with a dementia diagnosis were diagnosed as DAT probable and the 3 stable MCI and the single CN subject who converted to MCI were diagnosed as MCI due to DAT. One amnestic MCI patient was noted to have depression which required drug treatment and three patients had parkinsonian signs (their neuropathological diagnoses were AD + DLB, AD + DLB + MTL-TDP and AD + MTL-TDP). Finally, the patient with AD + SVD-I + AGD + MTL-TDP + HS had significant behavioral impairments.

**Figure 2:**
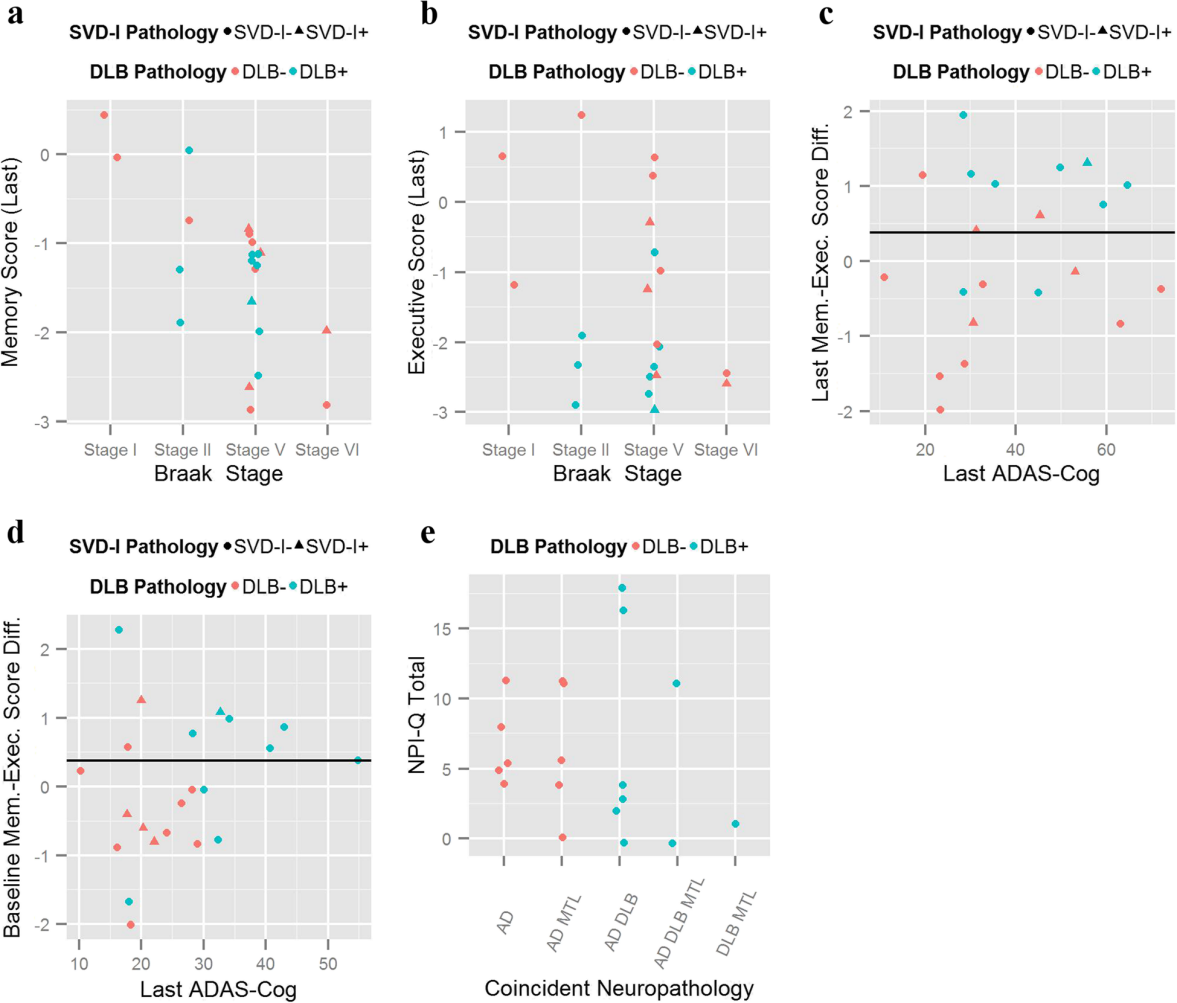
**Clinical correlates. a)** Memory and **b)** Executive summary composites in subjects stratified by Braak stage and presence of coincident DLB and SVD-I. **c)** Last and **d)** Baseline visit executive-memory mismatch based on the absence or presence of coincident DLB and/or SVD-I. **e)** NPI-Q total score based on neuropatholgically defined groups.

Whereas most of the subjects in the four groups were demented in their last visits before death, the degree of impairment varied between the groups at the baseline visit. Therefore, we compared neuropsychological performance at the last neuropsychological evaluation before death (median time between last visit and time of death 49.6 weeks, interquartile range 30.1-102.7 weeks) (Table 2). Higher Braak stages were associated with worse memory sum score (t = −3.04, p_adjusted_ = 0.026) (Figure 2a) and coincident DLB (t = −3.06, p_adjusted_ = 0.024) was associated with worse executive function (Figure 2b). No associations were found for SVD-I and/or MTL-TDP. Finally, we developed a mismatch score subtracting the executive summary score from the memory summary score. Therefore, subjects with a positive score would show predominant dysexecutive profile, whereas subjects with negative values have more severe memory impairment. The presence of coincident DLB was associated with a higher score (t = 3.7, p = 0.0015) and a mismatch cutoff score of 0.38 was associated with a sensitivity of 80.0% and a specificity of 83.3% to detect the presence of coincident DLB pathology (Figure 2c). When we analyzed the baseline visit the difference using the same cutoff and the cutoff retained its specificity but the sensitivity dropped (Sensitivity 70.0% and Specificity 83.3%) (Figure 2d).

**Table 2:** Association between neuropathological findings and neuropsychological and neuropsychiatric measures

	**Braak stage (I/II vs. V/VI)**	**Comorbid DLB**	**Comorbid MTL pathologies**	**Comorbid SVD-I**
Memory sum score	t = −3.01	t = 0.29	t = −1.64	t = 0.80
	p = 0.0070	p = 0.7795	p = 0.1198	p = 0.449
Executive function sum score	r = −0.34	t = 2.70	t = −0.76	t = 1.15
	p = 0.1326	p = 0.01423	p = 0.4553	p = 0.2922
NPI-Q	r = −0.33	t = 0.65	t = 0.70	t = −0.76
	p = 0.1632	p = 0.5239	p = 0.4928	p = 0.4553

P-values are adjusted for multiple comparisons.

There were no differences in the Neuropsychiatric Inventory Questionnaire (NPI-Q) total score based on the different neuropathological characteristics (Figure 2e, Table 2). Conversely, there was a strong association between the presence of hallucinations (item of the NPIQ-Q) and a coincident DLB (p = 0.0002); all four subjects who presented hallucinations in their last visit had a DLB (Sensitivity of 40% and specificity of 100%).

### CSF Biomarker findings

**Figure 3:**
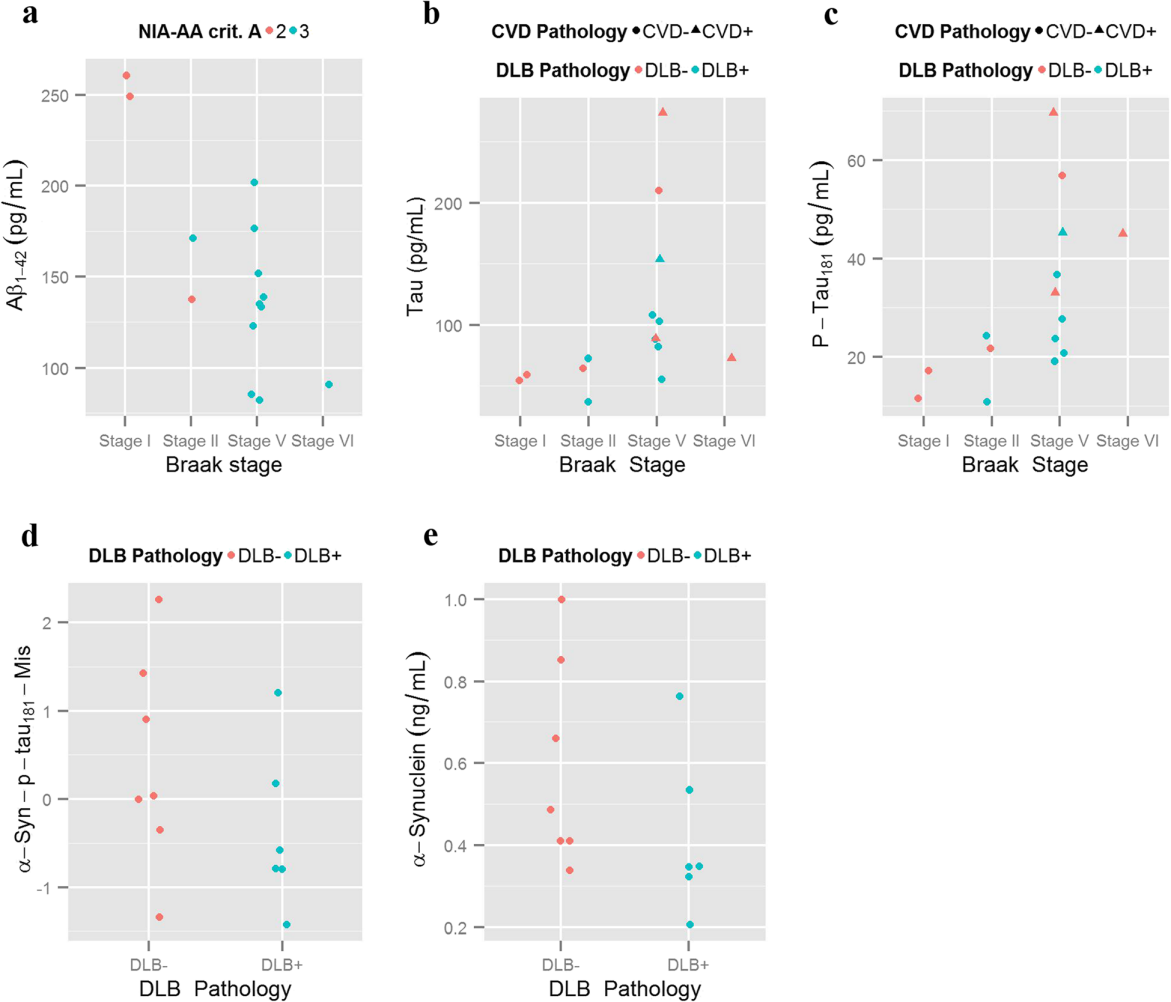
**CSF biomarker correlates. a)** Aβ_1-42_ levels based on a) NIA-AA criteria A score and Braak score. **b)**T-tau and **c)** P-tau_181_ levels based on Braak stage. **d)** α-Synuclein-p-tau_181_ mismatch and **e)** α-Synuclein based on presence or absence of coincident DLB and SVD-I.

CSF Aβ_1-42_, t-tau and p-tau_181_ measurements were available for 15 of the 22 deceased ADNI patients (Additional file 1: Table S3). As seen in Figure 3a all subjects had NIA-AA criteria A score of 2 or 3 using the recent NIA-AA criteria (For one subject Thal phase could not be estimated) [48]. Three subjects had CSF Aβ_1-42_ values equal or above 240 pg/mL (two of these subjects had a Braak stage I and the last one had a Braak stage II), whereas the remaining patients had values below 202 pg/mL. Both t-tau (r = 0.59, p = 0.0195) (Figure 3b) and p-tau_181_ (r = 0.70, p = 0.0035) (Figure 3c) correlated with Braak stage. Neither the presence of coincident DLB nor the presence of coincident MTL pathologies showed an association with CSF biomarker levels. 13 subjects had CSF α-synuclein measurements, but no differences were found for the α-synuclein p-tau181 mismatch (t = −1.31, p = 0.22) (Figure 3d) and α-synuclein (t = −1.59, p = 0.16) (Figure 3e) based on the presence or absence of DLB.

### Neuroimaging findings

Due to the small number of autopsied patients with amyloid neuroimaging PET scans, neither Pittsburgh B compound (PiB) PET nor AV45 PET scans were analyzed. On the other hand, 11 patients (Additional file 1: Table S3), 6 without DLB and 5 with comorbid DLB, had FDG-PET occipital lobe measures and 15 subjects had HCI measures available from FDG-PET performed at the baseline visit.

**Figure 4:**
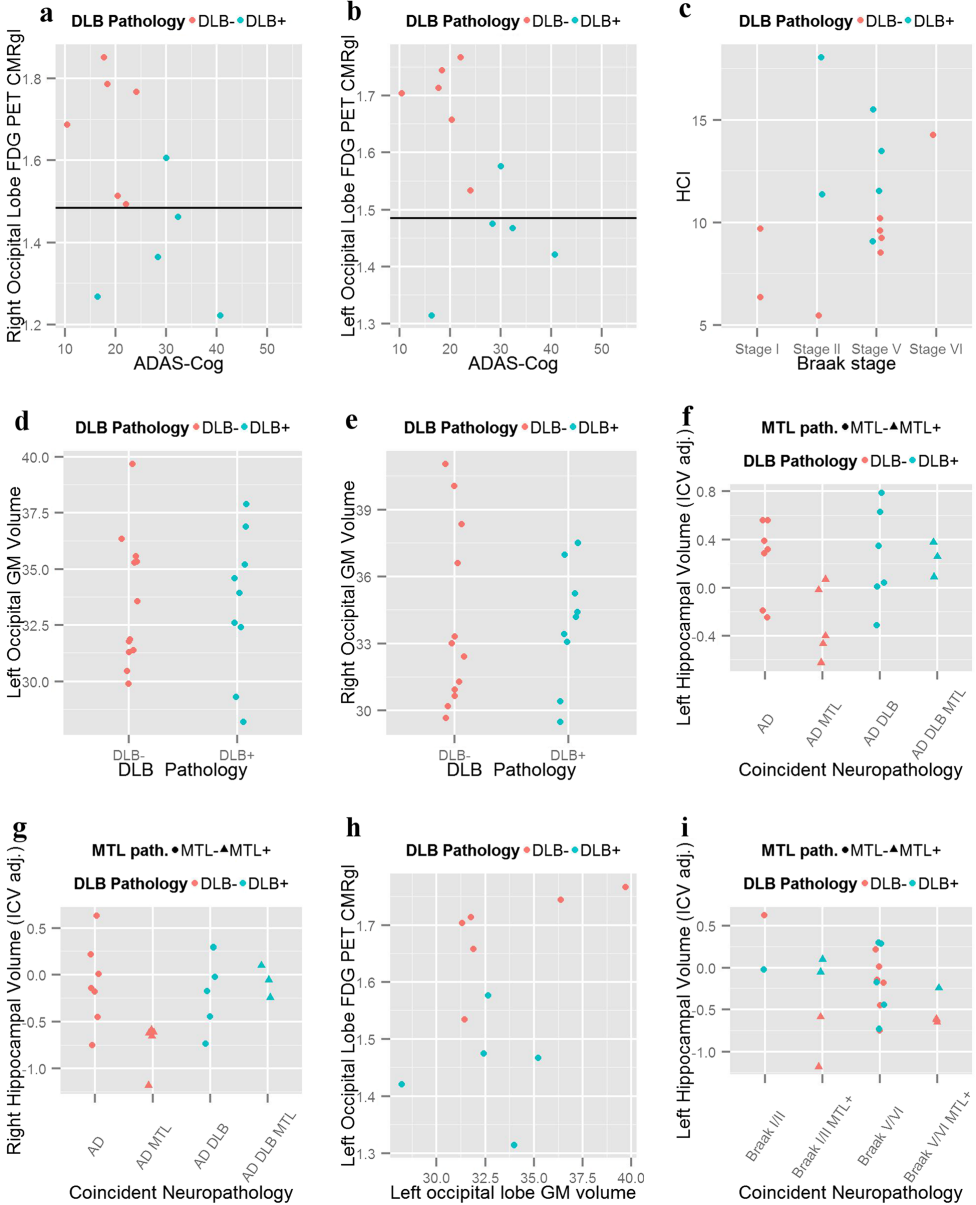
**Neuroimaging correlates. a)** Right and **b)** Left occipital lobe FDG PET CMRgI stratified by presence or absence of coincident DLB and their relationship to ADAS-Cog measure, **c)** FDG-PET HCI stratified by presence or absence of coincident DLB and its relationship to ADAS-Cog measure. **d)** Left and **e)** Right occipital GM volume stratified by presence or absence of coincident DLB. **f)** Left and **g)** Right hippocampal volume stratified by neuropathologically defined groups. **h)** Occipital lobe GM volume and FDG PET CMRgI. **i)** Left hippocampal volume stratified by Braak stage and coincident MTL pathology.

**Figure 5:**
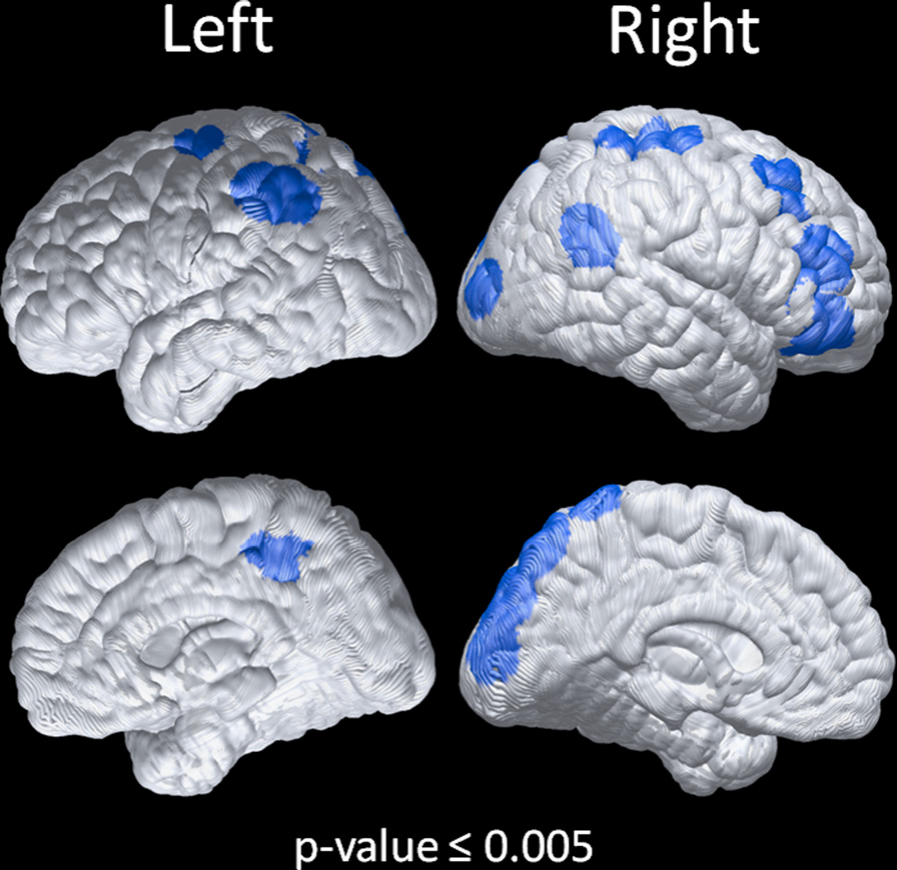
**Statistical cortical surface map for voxel-wise group comparison between subjects with and without DLB covarying out the effects of ADAS-Cog.** Brain areas of significant hypometabolism in DLB were shown with an uncorrected threshold of p ≤ 0.005.

Consistent with previous reports [21,25,27], DLB patients showed a significant right (t = −3.27, p = 0.0097) (Figure 4a) and left (t = −4.47, p = 0.0016) (Figure 4b) occipital lobe hypometabolism. After adjusting for ADAS-Cog score only the left occipital hypometabolism was significantly lower in the cases with DLB (right occipital metabolism: t = −2.21, p = 0.058; left occipital metabolism: t = −2.32, p = 0.041) and differences also remained significant after adjusting for time to death (right occipital metabolism: t = −3.78, p = 0.0054; left occipital metabolism: t = −4.67, p = 0.0016). The same cutoff value of 1.48 showed a sensitivity of 80% and a specificity of 100% to classify patients with DLB based on either the left or right occipital regions. Interestingly the AD-DLB case that was classified as AD had the lowest burden of LBs that were circumscribed to the amygdala, entorhinal cortex and midbrain, whereas all the other cases were diffuse neocortical cases. In addition, the presence of DLB (t = 2.54, p = 0.026), but not higher Braak stage (t = 0.78, p = 0.44), was associated with higher hypometabolic convergence index (HCI) values (Figure 4c). This association remained significant when the presence of a DLB diagnosis was further adjusted for Braak stage (t = 2.47, p = 0.031), the ADAS-Cog score at the time of scan (t = 2.32, p = 0.041) or time to death (t = 2.71, p = 0.020). Finally, we conducted a secondary voxel-wise group comparison covarying out ADAS-Cog score for disease severity and found several hypometabolic areas with coincident DLB as shown in Figure 5 and listed in the Additional file 1: Table S5

We studied two MRI areas selected *a priori* using the MRI gray matter (GM) volume adjusting for total intracranial volume (ICV): the occipital lobe, based on previous fluorodeoxyglucose (FDG)-PET findings, and the hippocampal volume, based on previous MRI findings. Neither left (t = 1.2, p = 0.24) or right (t = 1.48, p = 0.16) hippocampal volume, nor left (t = −0.09, p = 0.93) or right (t = 0.003, p = 1.0) occipital GM volume showed differences in subjects with coincident DLB in the baseline MRI (Figure 4d-g). Finally, we repeated the analysis of the MRI occipital and occipital GM volumes using different visits per subject based on a matched ADAS-Cog score (Additional file 1: Figure S1) and again found no group differences (Additional file 1: Table S4). In addition, there no correlation was found between the occipital GM volume and the occipital FDG CMRgl (left occipital lobe: r = 0.27, p = 0.42; right occipital lobe: r = 0.23, p = 0.50) (Figure 4h).

Finally, we tested the association of MTL-TDP on hippocampal volume. Because the neuropathologically studied hemisphere was the left one, we only examined the association with the left hippocampal volume using MRI scan acquired at the times with matched clinical severity. In the analysis that include Braak stage and presence of MTL-TDP there was a significant association between left hippocampal atrophy and MTL-TDP (t = −2.75, p = 0.013) and a trend with Braak stage (t = −1.86, p = 0.080) (Figure 4i).

### Prediction of DLB pathology in the ADNI cohort

A total of 76 DAT patients and 139 MCI ADNI-1 subjects had occipital lobe hypometabolism measures and 39.5% and 11.5% of them had occipital hypometabolism below the 1.48 cutoff, respectively. Of the 193 ADNI-1 DAT subjects a total of 30.1% had a predominantly executive cognitive impairment which would be characteristic of non-DAT pathology.

## Discussion

We performed a multimodal biomarker analysis of consecutive ADNI subjects who came to autopsy after longitudinal follow-up to death. Most of the subjects were late amnestic MCI and probable DAT at baseline visit, without any atypical clinical presentation. In addition, subjects with vascular disease or a Hachinski Ischemic Score >4 were excluded from the ADNI study [29]. Despite this, only four out of 22 subjects had a neuropathological diagnosis of pure AD (13.6%), while DLB, MTL-TDP and infarcts were present in 45.5%, 40.9% and 22.7% of the patients, respectively. A predominantly executive dysfunction was associated with the presence of DLB and hallucinations, as recorded in the NPI-Q. CSF fluid Aβ_1-42_, t-tau and p-tau_181_ were associated with the phosphorylated tau (neurofibrillary tangle) Braak stage and the NIA-AA criteria A score, but not with other pathologies. Baseline occipital hypometabolism accurately predicted the presence of DLB pathology, whereas MRI GM occipital atrophy did not. MTL-TDP and AGD were associated with greater hippocampal atrophy.

Despite the neuropathological heterogeneity of the patients, all the demented subjects had a DAT probable clinical diagnosis and the MCI patients had and AD-like profile; three subjects had parkinsonian signs, of those two had DLB pathology and one did not. Therefore, an overall classical DAT presentation does not rule out the presence of coincident vascular disease or other neurodegenerative disease even in the presence of CSF and PET amyloid imaging findings compatible with AD pathology. This is not surprising because memory impairment is the most common presenting clinical symptom of clinically diagnosed DLB (cDLB) patients (with confirmed abnormal dopamine transporter imaging) [49] and most of the DLB/AD + DLB cases were diagnosed as DAT, showing a low sensitivity of the clinical criteria, at least in this small series [50]. One difference between our study and other previous studies is that we did not include any patients with a cDLB diagnosis and patients with coincident DLB in our study had a typical DAT profile. Nevertheless, we found two clinical markers that were highly predictive of coincident pathologies: a predominant dysexecutive syndrome and the presence of hallucinations. A previous neuropathological study found a high specificity of visual hallucinations in DLB (with a prevalence of 1% early in the course of AD), although prevalence of visual hallucinations was low and therefore not was not a sensitive biomarker [51]. In our study, we found that during the progression of disease a prominence of dysexecutive impairment in the presence of an amnestic profile is a marker for coincident DLB. This is consistent with a previous study that described a worse executive function in subjects with DLB and worse memory in patients with AD, although no classification performance was reported [52]. However, a predominantly disexecutive syndrome might not be a specific biomarker and other NDDs like frontotemporal lobar degeneration could have a similar profile.

Currently, Aβ amyloid PET imaging and CSF Aβ_1-42_ are the most widely accepted research biomarkers for AD which have shown an important correlation with brain Aβ amyloid deposition [20,26,53] and with each other [54]. Confirming the results of our previous study in which one fourth of the patients had coincident pathologies [14], mainly DLB, and studies for other groups [19], CSF Aβ, t-tau and p-tau_181_ levels can reliably predict AD pathology even in the presence of other coincident pathologies and subjects with Aβ levels above the published cutoff [34] had a low burden of AD.

Three studies with FDG-PET and several neuropathologically confirmed cases have previously reported occipital FDG-PET hypometabolism independent of coincident AD: Albin et al. included three DLB and three with AD + DLB [25], Kantarci et al. included 2 AD and 3 DLB (and a larger number of clinically diagnosed cases) [21] and Minoshima et al. included 7 AD + DLB, 4 DLB and 10 AD (and a larger number of clinically diagnosed cases) [27]. The last study reported a sensitivity of 90% and a specificity of 80% for the diagnosis of DLB with or without AD based on hypometabolism in the occipital cortex [27]. Kantarci et al. carried out a study mostly based on clinically diagnosed patients, and they described an area under the curve (AUC) in the receiver operating characteristic (ROC) of 0.84 for the FDG-PET with cDLB patients showing an occipital hypometabolism independently of Aβ deposition measured by PiB PET [21]. In our study, we found an 80% sensitivity and a 100% specificity based on occipital FDG-PET hypometabolism. All of our DLB subjects had coincident AD and this did not affect the accuracy of the classification. In addition, the only DLB case that was classified as non DLB by the occipital FDG-PET cutoff was the only one that did not have diffuse neocortical LBs. The percentage of predicted DLB pathology in the ADNI-1 DAT subjects was similar to the one observed in the autopsied subjects. Interestingly, it has been suggested that occipital hypometabolism might be an preclinical biomarker of DLB [55]. On the other hand, functional neuroimaging approaches that measure striatal dopaminergic innervation and myocardial sympathetic nerve integrity might be more specific to changes associated with LB pathology, specially the latter which captures postganglionic denervation which is present in PD and DLB patients, but not in patients with other atypical parkinsonisms [56]. However, doing these additional tests would increase the, cost, time and inconvenience for patients, whereas FDG PET is also helpful for the differential diagnosis in non-parkinsonian syndromes. Therefore a FDG PET measure that specifically predicts coincident DLB pathology would be preferable. The voxels that contribute to HCI are located in temporal, occipital and parietal cortices and it might be possible that the association with HCI with the presence of DLB and not Braak stage might reflect that this measure is also capturing the posterior cortical metabolism characteristic of DLB. Nevertheless, the AD group without DLB was less impaired at baseline and we did not analyze the FDG PET scans that were matched for clinical severity.

Neither our study nor others have found occipital lobe atrophy in cDLB or DLB [21,23]. In addition, we found no differences in hippocampal volume based on the presence of coincident DLB. Conversely, it has been reported that cDLB patients have similar hippocampal volume as CN subjects but lower hippocampal volume than DAT patients with an high diagnostic accuracy [21] and that a semiquantitative visual rating of MTL MRI atrophy had a high accuracy to classify AD against DLB and pathologically diagnosed vascular cognitive impairment patients [24]. The definition of the DLB group might explain these differences. For example, the study by Burton et al. included cDLB diagnosis patients [24] and the multimodal study by Kantarci et al. was mostly comprised of cDLB patients [21], therefore it can be expected that the pattern of atrophy is different in DLB with a cDLB diagnosis compared to those AD + DLB with a DAT diagnosis. In addition, the study by Kantarci et al. described a large sample of neuropathologically diagnosed subjects in whom only DLB subjects with high DLB probability as defined by McKeith criteria [46] had similar hippocampal volume as CN subjects, whereas intermediate and low probability DLB had similar hippocampal atrophy as AD subjects [22]. Therefore, hippocampal volume might not be a good marker of coincident DLB in a cohort of DAT subjects.

Late MCI ADNI 1 patients and DAT patients recruited in ADNI represent amnestic, cognitively impaired, subjects without any cognitive signs or symptoms suggestive of non-AD pathologies and a low vascular risk profile [29]. Therefore, these patients with multiple coincident pathologies represent the typical patients recruited in AD clinical trials. These multiple coincident pathologies have important implications for clinical trials and the approach for treating patients. Studies of the brains of patients treated with Aβ immunotherapy have shown a decrease of total Aβ burden and a decrease of neurite curvature ratio and a [57-59] and an increase of amyloid deposition in neocortical blood vessels that might decrease over time [59], without any strong effect on tau [57] or α-synuclein [60] clearance. Therefore, multimodal biomarker approaches that aim to detect the different coincident pathologies (instead to categorizing patients into a single diagnostic category), will be needed to select homogenous populations for protein-specific targeted clinical trials and to tailor the treatment for each patient.

## Conclusions

In summary, our study of longitudinally assessed participants in ADNI who had biomarker and postmortem neuropathology found the clinical diagnosis of DAT and MCI due to AD was supported by a pathologic diagnosis of AD in all cases, although some cases had low burden of AD neuropathologic changes. Although this is a small sample, this observation is consistent with the concept that the clinical spectrum of AD represents a continuous process. A large proportion of the ADNI AD cases had heterogeneous comorbidities. Whereas CSF AD biomarkers might be able to predict AD pathology additional biomarkers (neuropsychological profile and FDG-PET) were useful to predict coincident DLB and therefore the combination is useful to predict both pathologies. Hippocampal volume was not a useful biomarker to predict coincident pathologies in this type of patients. These data have implications for the development of biomarkers which are specific for coincident pathologies in addition to AD and are able to establish the diagnosis of the different pathologies present in the subject. Finally, the presence of frequent comorbidities may help to explain variance in biomarker, structural and functional imaging data and be of utility in explaining altered responses to proposed therapeutic interventions.

## Competing interests

M.W.W.: stock options, Elan, Synarc; travel expenses, Novartis, Tohoku University, Fundacio Ace, Travel eDreams, MCI Group, NSAS, Danone Trading, ANT Congress, NeuroVigil, CHRU-Hopital Roger Salengro, Siemens, AstraZeneca, Geneva University Hospitals, Lilly, University of California, San Diego–ADNI, Paris University, Institut Catala de Neurociencies Aplicades, University of New Mexico School of Medicine, Ipsen, Clinical Trials on Alzheimer’s Disease, Pfizer, AD PD meeting, Paul Sabatier University; board membership, Lilly, Araclon, Institut Catala de Neurociencies Aplicades, Gulf War Veterans Illnesses Advisory Committee, VACO, Biogen Idec, Pfizer; consultancy, AstraZeneca, Araclon, Medivation = Pfizer, Ipsen, TauRx Therapeutics, Bayer Healthcare, Biogen Idec, ExonHit Therapeutics, Servier, Synarc, Pfizer, Janssen; honoraria, NeuroVigil, Insitut Catala de Neurociencies Aplicades, PMDA = Japanese Ministry of Health, Labour, and Welfare, Tohoku University; commercial research support, Merck, Avid; government research support, DOD, VA. Other authors report no conflicts of interest. JM has consulted for Eisai, Glaxo-SmithKline, Novartis, Estavo Jansen Alzheimer Immunotherapy Program, Pfizer and Eli Lilly/Avid Radiopharmaceuticals. D. Aisen serves on a scientific advisory board for NeuroPhage and as a consultant to Elan Corporation, Wyeth, Eisai Inc., Bristol-Myers Squibb, Eli Lilly and Company, NeuroPhage, Merck & Co., Roche, Amgen, Abbott, Pfizer Inc, Novartis, Bayer, Astellas, Dainippon, Biomarin, Solvay, Otsuka, Daiichi, AstraZeneca, Janssen, Medivation, Inc., Theravance, Cardeus, and Anavex and receives research support from Pfizer Inc., Baxter International Inc.

## Authors’ contributions

All authors read and approved the final manuscript, contributed to interpretation of the data and critical review of the manuscript and study concept. NJC and JCM established the neuropathological diagnoses and gradings. DC and EH collected and performed immunohistochemistry. XD and CD processed and analyzed the MRI data. KC, AF, NA, AR, RJB and EMR processed and analyzed the FDG-PET data. JBT drafted the manuscript and performed the statistical analyses. JQT drafted the manuscript.

## Supplementary Material

Additional file 1 **Supplementary Material.** Clinical and Multimodal Biomarker Correlates of ADNI Neuropathological Findings. Click here for file
