# Supplementary material for: Clinical and multimodal biomarker correlates of ADNI neuropathological findings
Source: Acta Neuropathol Commun. 2013 Oct 9;1:65. doi: 10.1186/2051-5960-1-65 (PMC3893373; doi:10.1186/2051-5960-1-65)
Supplement: Additional file 1 — Supplementary Material. Clinical and Multimodal Biomarker Correlates of ADNI Neuropathological Findings. [file 2051-5960-1-65-S1.docx]

**Supplementary Material: Clinical and Multimodal Biomarker Correlates of ADNI Neuropathological Findings**

Jon B. Toledo*, Nigel J. Cairns*, Xiao Da, Kewei Chen, Deborah Carter, Adam Fleisher , Erin Householder, Napatkamon Ayutyanont, Auttawut Roontiva, Robert J Bauer, Paul Eisen, Leslie M. Shaw, Christos Davatzikos, Michael W. Weiner, Eric Reiman, John Morris** and John Q. Trojanowski^a^**, for the Alzheimer’s Disease Neuroimaging Initiative

**Methods**

ADNI description and goals

The primary goal of ADNI is to test whether serial MRI [[1](#_ENREF_1), [2](#_ENREF_2)], PET [[3](#_ENREF_3)], other biological markers [[4](#_ENREF_4)], and clinical and neuropsychological assessment [[5](#_ENREF_5)] can be combined to measure the progression of MCI and early AD. Determination of sensitive and specific markers of very early AD progression is intended to aid researchers and clinicians to develop new treatments and monitor their effectiveness, as well as lessen the time and cost of clinical trials. The Principal Investigator of this initiative is Michael W. Weiner, MD, VA Medical Center and University of California – San Francisco, while Les Shaw and John Trojanowski at the University of Pennsylvania lead the Biomarker Core and John Morris and Nigel Cairns at Washington University lead the Neuropathology Core. ADNI is the result of efforts of many co-investigators from a broad range of academic institutions and private corporations.

Recruitment inclusion and exclusion criteria for ADNI 1

Inclusion criteria were as follows: 1) Hachinski Ischemic Score ≤4; 2) Permitted medications stable for 4 weeks prior to screening; 3) Geriatric Depression Scale score < 6; 4) visual and auditory acuity adequate for neuropsychological testing; good general health with no diseases precluding enrollment; 5) 6 grades of education or work history equivalent; 6) Ability to speak English or Spanish fluently; 7) A study partner with 10 hours per week of contact either in person or on the telephone and who could accompany the participant to the clinical visits.

Criteria for the different diagnostic groups are summarized in supplementary table 1. Groups were age matched. Cognitively (CN) subjects could not have any significant cognitive impairment or impaired activities of daily living. Clinical diagnosed Alzheimer’s disease patients (cAD) had mild cAD and had to meet the National Institute of Neurological and Communicative Disorders and Stroke–Alzheimer’s Disease and Related Disorders Association criteria for probable AD [[6](#_ENREF_6)], whereas mild cognitive impairment subjects (MCI) should not meet this criteria and have largely intact general cognition and functional performance.

*CSF Biomarker collection and analysis*

CSF was collected into polypropylene collection tubes or syringes provided to each site, then transferred into polypropylene transfer tubes without any centrifugation step followed by freezing on dry ice within 1 hour after collection, and shipped overnight to the ADNI Biomarker Core laboratory at the University of Pennsylvania Medical Center on dry ice. Aliquots (0.5 ml) were prepared from these samples after thawing (1 hour) at room temperature and gentle mixing. The aliquots were stored in bar code–labeled polypropylene vials at -80°C. Fresh, never before thawed 0.5 mL aliquots for each subject’s set of longitudinal time points were analyzed on the same 96 well plate in the same analytical run for this study in order to minimize run to run and reagent kit lot sources of variation. Within run coefficient of variation (%CV) for duplicate samples ranged from 2.5-5.9% for Aβ_1-42_, 2.2-6.3% for t-tau and 2.0-6.5% for p-tau_181_ and the inter-run %CV for CSF pool samples ranged from 5.1-14% for Aβ_1-42_, 2.7 -11.2% for t-tau and 3.3-11.5% for p-tau_181_. Further information on the procedures and standard operating procedures (SOP) can be found in previous publications [[4](#_ENREF_4), [7](#_ENREF_7)] and online (<http://www.adni-info.org/>). The α-syn luminex assay applied in the ADNI demonstrated low day-to-day as well as plate-to-plate signal variability (< 5% with > 80 plates analyzed), with high signal reproducibility and linear performance in the low pg range (sensitivity ranged as low as 0.009 ng/ml). The accuracy for the assay, as determined by recovery of spike α-synuclein protein, was approximately 93 %. A detailed protocol can be downloaded from the ADNI website.

**Results**

Comparisons of deceased patients to diseased patients without autopsy and the whole ADNI-1 cohort

Because ADNI-GO and ADNI-2 have different inclusion criteria for the MCI subjects and the follow-up is shorter and all except one of the deceased autopsied patients belonged to the ADNI-1 cohort we limited our comparisons to patients who were recruited in ADNI-1. Results are summarized in supplementary table 2.

**Supplementary table 1**. ADNI 1 criteria for recruitment of CN, MCI and AD subjects.

|  | CN | MCI | AD |
| --- | --- | --- | --- |
| Memory complaints | Absent | Present | Present |
| MMSE | 24-30 | 24-30 | 20-26 |
| CDR | 0 | 0.5^1^ | 0.5-1.0 |
| Delayed recall Logical Memory II subscale of WMSR | 16 YoEd: ≥9  8–15 YoEd: ≥5  0-7 YoEd: ≥3 | 16 YoEd: ≤8  8–15 YoEd: ≤4  0-7 YoEd: ≤2 | 16 YoEd: ≤8  8–15 YoEd: ≤4  0-7 YoEd: ≤2 |

AD: Alzheimer’s disease; CDR: Clinical Dementia Rating; CN: Cognitively normal; MCI: Mild cognitive impairment; MMSE: Mini-Mental State Examination; YoEd : years of education. ^1^Mandatory requirement of the memory box score being 0.5 or greater.

**Supplementary table 2**. Characteristics of ADNI-1 subjects who died, with and without autopsy, and subjects who are not known to be deceased.

|  | Dead with autopsy  (n=21) | Dead without autopsy  (n=41) | p-value^3^ | Subjects not known to be dead  (n=757) | p-value^4^ |
| --- | --- | --- | --- | --- | --- |
| Age at baseline^1^ | 77.4 (6.5) | 77.9 (7.3) | 0.79 | 75.0 (6.8) | 0.11 |
| Clinical diagnosis at baseline | 4.8% CN  52.4% MCI  42.9% AD | 24.4% CN  61.0% MCI  14.6% AD | 0.025 | 28.8% CN  47.7% MCI  23.5% AD | 0.014 |
| Gender (n male/total) | 16/21 | 34/41 | 0.52 | 427/757 | 0.11 |
| Education (years) ^1^ | 15.2 (2.4) | 15.5 (3.2) | 0.78 | 14.0 (2.3) | 0.65 |
| APOE ε4 (n positive/total) | 11/21 | 18/41 | 0.71 | 371/757 | 0.93 |
| ADAS-Cog (13 item) Baseline ^2^ | 24.0 (18.-30.0) | 17.7 (10.3-23.0) | 0.002 | 17.7 (11.0-24.0) | 0.002 |

**Supplementary Table 3.** Neuropathological findings and available biomarkers in the studied subjects.

| RID | Group | Clinical Diagnosis | Primary neuropth. diagnosis | Secondary neuropth. Diagnoses | Braak Stage | Neuritic Plaques | NIA-AA criteria A score ^1^ | LB Stage | CAA | Athero-sclerosis | CSF | FDG PET |
| --- | --- | --- | --- | --- | --- | --- | --- | --- | --- | --- | --- | --- |
| 78 | AD | AD | AD |  | VI | Freq. | 3 | No LB | Moderate | Severe | Yes | - |
| 362 | AD | MCI to AD | AD | SVD-I | V | Freq. | 3 | No LB | Moderate | Severe | Yes | Occ.  HCI |
| 400 | AD | AD | AD | SVD-I | VI | Freq. | 3 | No LB | Moderate | Severe | - | HCI |
| 458 | AD | MCI | AD |  | V | Freq. | 3 | No LB | Mild | Mild | - | - |
| 821 | AD | MCI | AD |  | V | Freq. | 3 | No LB | Mild | Mild | - | - |
| 1203 | AD | CN to MCI | AD |  | I | No | 2 | No LB | Moderate | None | Yes | Occ.  HCI |
| 1425 | AD | MCI to AD | AD | SVD-I | V | Freq. | 3 | No LB | Mild | Severe | - | HCI |
| 326 | AD+ MTL-TDP | MCI to AD | AD | AGD+ MTL-TDP | II | Sparse | NA | No LB | Mild | Mild | Yes | Occ.  HCI |
| 853 | AD+ MTL-TDP | AD | AD | AGD+ MTL-TDP+HS | V | Freq. | 3 | No LB | Mild | Mild | - | - |
| 880 | AD+MTL-TDP | MCI | AD | AGD+ MTL-TDP | I | Sparse | 2 | No LB | Mild | Mild | Yes | Occ.  HCI |
| 1282 | AD+ MTL-TDP | MCI to AD | AD | MTL-TDP | V | Freq. | 3 | Amygdala predominant | Mild | Mild | Yes | Occ.  HCI |
| 1393 | AD+MTL-TDP | MCI to AD | AD | SVD-I+AGD+ MTL-TDP+HS | V | Freq. | 3 | No LB | Moderate | Mild | Yes | Occ.  HCI |
| 492 | AD+DLB | AD | AD |  | V | Freq. | 3 | LB unspecified | Mild | Mild | Yes | Occ.  HCI |
| 565 | AD+DLB | AD | AD |  | V | Freq. | 3 | LB Diffuse | Moderate | Mild | Yes | Occ.  HCI |
| 691 | AD+DLB | AD | DLB |  | II | No | 2^2^ | LB Diffuse | None | NA | - | - |
| 724 | AD+DLB | AD | AD | SVD-I | V | Freq. | 3 | LB Diffuse | Moderate | Mild | - | - |
| 834 | AD+DLB | MCI to AD | AD |  | V | Freq. | 3 | LB Diffuse | Mild | Mild | - | HCI |
| 916 | AD+DLB | AD | AD |  | V | Freq. | 3 | LB Diffuse | Mild | Mild | - | - |
| 53 | DLB+ MTL-TDP | AD | DLB | AD+  MTL-TDP | II | No | 2 | LB Diffuse | Mild | None | - | Occ.  HCI |
| 567 | AD+DLB+ MTL-TDP | MCI to AD | AD | MTL-TDP | V | Freq. | 3 | LB Diffuse | Mild | Mild | Yes | Occ.  HCI |
| 723 | AD+DLB+ MTL-TDP | MCI to AD | DLB | AGD | II | Sparse | 3 | LB Diffuse | Moderate | Mild | Yes | Occ.  HCI |
| 4223 | AD+DLB+ MTL-TDP | AD | AD | MTL-TDP | V | Freq. | 3 | LB Diffuse | Moderate | None | - | HCI |

^1^ Thal phase summarized as indicated for A (Aβ) stage of the NIA-AA AD criteria (scale 1-3)*;[[8](#_ENREF_8)] This case was missing amyloid staining for pons, medulla and cerebellum.

CAA: Cerebral amyloid angiopathy; NA: Not available; Occ.: Occipital lobe FDG PET measure available.

**Supplementary Table 4.** Occipital and hippocampal GM volume comparison based on the presence of coincident DLB in ADAS-Cog matched subjects.

|  | Presence of DLB |
| --- | --- |
| Occipital right GM | t=-0.252  p=0.80 |
| Occipital left GM | t=0.068  p=0.95 |
| Right hippocampus | t=1.37  p=0.19 |
| Left hippocampus | t=1.39  p=0.18 |

**Supplementary Table 5.** Areas with significant hypometabolism in subjects with coincident DLB.

| Area | Hemisphere | X | Y | Z | t-value | p-value |
| --- | --- | --- | --- | --- | --- | --- |
| Angular gyrus | Right | 51 | -53 | 25 | 3.43 | 0.0025 |
| Calcarine | Right | 20 | -83 | 8 | 3.74 | 0.0014 |
| Cuneus | Left | -4 | -92 | 30 | 4.53 | 0.0003 |
| Inferior frontal gyrus | Right | 42 | 27 | -1 | 5.54 | 0.0001 |
| Middle frontal gyrus | Left | -32 | 15 | 58 | 3.97 | 0.0009 |
| Middle temporal gyrus | Right | 51 | -51 | 21 | 4.2 | 0.0006 |
| Orbitofrontal cortex | Right | 46 | 40 | -9 | 3.75 | 0.0014 |
| Paracentral lobule | Right | 14 | -35 | 46 | 3.88 | 0.0011 |
| Postcentral gyrus | Left | -36 | -34 | 64 | 3.91 | 0.0010 |
| Precentral gyrus | Right | 42 | -22 | 64 | 4.71 | 0.0003 |
| Precuneus | Right | 14 | -44 | 43 | 5.09 | 0.0001 |
| Superior occipital lobe | Left | -8 | -79 | 48 | 3.76 | 0.0014 |
| Superior parietal lobe | Left | -26 | -52 | 47 | 4.02 | 0.0008 |
| Inferior parietal lobe | Left | -59 | -41 | 43 | 3.96 | 0.0009 |

Uncorrected p-values. Coordinates based on Talairach atlas.

**Supplementary Figure 1.** ADAS-Cog scores corresponding to the visits of the MRI matched for severity and stratified by presence of DLB and MTL (AGD, MTL-TDP and HS) pathologies.


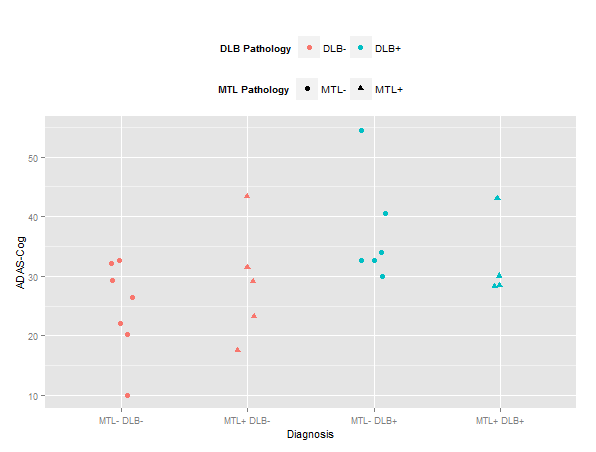


**References**

1. Jack CR, Jr., Bernstein MA, Fox NC, Thompson P, Alexander G, Harvey D, Borowski B, Britson PJ, J LW, Ward C, et al: **The Alzheimer's Disease Neuroimaging Initiative (ADNI): MRI methods.** *Journal of magnetic resonance imaging : JMRI* 2008, **27:**685-691.

2. Weiner MW, Veitch DP, Aisen PS, Beckett LA, Cairns NJ, Green RC, Harvey D, Jack CR, Jagust W, Liu E, et al: **The Alzheimer's Disease Neuroimaging Initiative: a review of papers published since its inception.** *Alzheimer's & dementia : the journal of the Alzheimer's Association* 2012, **8:**S1-68.

3. Jagust WJ, Bandy D, Chen K, Foster NL, Landau SM, Mathis CA, Price JC, Reiman EM, Skovronsky D, Koeppe RA: **The Alzheimer's Disease Neuroimaging Initiative positron emission tomography core.** *Alzheimer's & dementia : the journal of the Alzheimer's Association* 2010, **6:**221-229.

4. Shaw LM, Vanderstichele H, Knapik-Czajka M, Clark CM, Aisen PS, Petersen RC, Blennow K, Soares H, Simon A, Lewczuk P, et al: **Cerebrospinal fluid biomarker signature in Alzheimer's disease neuroimaging initiative subjects.** *Annals of neurology* 2009, **65:**403-413.

5. Petersen RC, Aisen PS, Beckett LA, Donohue MC, Gamst AC, Harvey DJ, Jack CR, Jr., Jagust WJ, Shaw LM, Toga AW, et al: **Alzheimer's Disease Neuroimaging Initiative (ADNI): clinical characterization.** *Neurology* 2010, **74:**201-209.

6. McKhann G, Drachman D, Folstein M, Katzman R, Price D, Stadlan EM: **Clinical diagnosis of Alzheimer's disease: report of the NINCDS-ADRDA Work Group under the auspices of Department of Health and Human Services Task Force on Alzheimer's Disease.** *Neurology* 1984, **34:**939-944.

7. Shaw LM, Vanderstichele H, Knapik-Czajka M, Figurski M, Coart E, Blennow K, Soares H, Simon AJ, Lewczuk P, Dean RA, et al: **Qualification of the analytical and clinical performance of CSF biomarker analyses in ADNI.** *Acta neuropathologica* 2011, **121:**597-609.

8. Hyman BT, Phelps CH, Beach TG, Bigio EH, Cairns NJ, Carrillo MC, Dickson DW, Duyckaerts C, Frosch MP, Masliah E, et al: **National Institute on Aging-Alzheimer's Association guidelines for the neuropathologic assessment of Alzheimer's disease.** *Alzheimer's & dementia : the journal of the Alzheimer's Association* 2012, **8:**1-13.
